# Practical Notes and Formulæ

**Published:** 1881-05-20

**Authors:** 


### PRACTICAL NOTES AND FORMULÆ.

**Unknown Eruptive Diseases.**—Dr. J. S. Knott, of Texas, writes: We have had in our town and vicinity, an eruptive disease resembling measles. Some of the cases have huskiness of voice, suffusion of the eyes, etc., and many of the persons so afflicted had measles when young. Two infants under my care had same eruption, who had no opportunity to get the disease from other persons. I will advise you of one case in particular, and give you my treatment, and let you name the disease for me. My medical friends in town claim it measles, but I can't think so of the cases under my care; it is I believe admitted by nearly all writers (medical) that persons have been known to have measles twice, but very seldom. In one family in town, all the children in the family except one, born in Texas, had measles in Tennessee, and when this disease came among us, those in that particular family that had measles previously, were the first to break out with this affection.

*A Case in Point.*—I was called about dark to see Mrs. C., *enciente;* found pulse quick, skin hot, vomiting almost incessantly; she had kept nothing on the stomach for a day or two. Her husband claimed to be something of a doctor himself—told me he could not quiet the stomach by using any means. She had eaten nothing; no appetite for two or three days. My prescription which I made at once, was eng. calomel, nit. bismuth, with small portion morphine sulph. I gave her first dose at 8 o'clock, placed it on the tongue dry, and with swallow of water was taken without any trouble. She rested quietly until near the time to take second dose same as first, when I had her taken off the bed and given a good hip bath, when the other powder was taken. She expressed herself much better the following morning, got a good action from the liver and she was well. That was the only medicine I gave her except two or three small doses of bismuth, which we had no occasion to use after the bowels moved. I also used tinct. val. and bromide to procure rest, but she never vomited after taking first dose calomel. In two or three days she was up, and her husband had almost despaired of her living at all. Now is it measles? If so I never knew before that two doses calomel would cure measles at once.

I will further state Mrs. C. had sore throat in connection with the eruption, and her child two years old was similarly affected, or had same symptoms, and I also gave her a mercurial course same night, and the child never had the eruption at all.

[In the cases above referred to the symptoms are not given in sufficient detail or accuracy to enable us to name the disease of which our friend writes, but so far as we can form an opinion from what he has written, we think the disease is *Rotheln*—a form of eruption resembling measles, though milder in character, and as the throat is not unfrequently affected, may be mistaken for scarlatina. Catarrhal symptoms are generally absent, but should a cold chance to co-exist the likeness to measles is very strong. The disease, however, usually terminates about the time that measles is at its height (4th or 5th day). The systemic disturbance is usually much less than measles, and the temperature never high unless there be some complication to aggravate the case. It usually prevails epidemically, and if contagious at all only moderately so.—ED. W.]

**Diphtheria.**—Dr. R. S. Forehand, of Georgia, suggests the following treatment for diphtheria: Irritate the throat from ear to ear with croton oil, and then make a mouth-wash or gargle for the throat, 20 drops of creasote to 5 ounces of good apple vinegar, mix and shake well. Wash the mouth and gargle the throat every three or four hours. If secretions have been swallowed, give castor oil to open bowels, or give an emetic of ipecac. Do not use milk for diet, but use wine freely. When convalescent give tincture of iron for a few days as a tonic.

**Startin's Mixture.**—A wonderfully valuable combination of sulphur is that known as "Startin's Mixture:"

| | | | |
|---|---|---|---|
| ℞ Magnes. sulph | ℥j, | 32.00 | Gm. |
| Ferri sulph | ʒj, | 4.00 | " |
| Acid sulph. dil | ʒij, | 8.00 | " |
| Tinct. gentian | ℥j, | 32.00 | " |
| Aquæ | ℥iij | 96.00 | " |

M. Sig. One ounce (thirty-two grams) dose after meals.

This is very potent in reducing cutaneous congestion in such conditions as erythema multiforme, erythematous eczema, and urticaria.—*Canada Lancet.*

**Gold Medal Cologne.**—

| | | |
|---|---|---|
| Oil of bergamot | 7 | parts. |
| Oil of citron, [cedrat] | 17 | " |
| Oil of neroli, petale | 10 | " |
| Oil of neroli, bigarade | 3-5 | " |
| Oil of rosemary, | 7 | " |
| Wine alcohol | 3000 | " |

The wine alcohol may be replaced with the spirit known here as Cologne alcohol.—*Chemist and Druggist.*

**Dabell's Purgative Tincture.**—

| | |
|---|---|
| ℞ Res. podophylli | gr. ij. |
| Essentiæ zingiber | ʒ j. |
| Spts. vin. recti | ℥ j. |

M. S. One drachm at night when lying down every two or three nights. Podophyllin is claimed to act mildly.—*Gillard's Med. Journal.*

**Remedies for Poison Oak Eruptions.**—Sulphate of soda, two drachms, chloral hydrate, one drachm, water, one pint. Mix. Use as a wash. It relieves in a very few hours.—*Eclectic Medical Journal.*

Sweet oil, 1 ounce; carbolic acid, 15 drops; ammonia, 10 drops. Mix. Apply freely to the affected parts. There will be no sign of the eruption in 48 hours.—*Ibid.*

